# Changes in the Fatty Acid Composition of Milk of Lipizzaner Mares during the Lactation Period

**DOI:** 10.3390/metabo12060506

**Published:** 2022-05-31

**Authors:** Maja Gregić, Pero Mijić, Mirjana Baban, Jasna Aladrović, Lana Pađen, Vesna Gantner, Tina Bobić

**Affiliations:** 1 Department of Animal Production and Biotechnology, Faculty of Agrobiotechnical Sciences Osijek, University of J. J. Strossmayer Osijek, V. Preloga 1, 31000 Osijek, Croatia; mgregic@fazos.hr (M.G.); mbaban@fazos.hr (M.B.); vgantner@fazos.hr (V.G.); tbobic@fazos.hr (T.B.); 2 Department of Physiology and Radiobiology, Faculty of Veterinary Medicine University of Zagreb, Heunzelova 55, 10000 Zagreb, Croatia; jasna.aladrovic@vef.unizg.hr (J.A.); lana.padjen@vef.unizg.hr (L.P.)

**Keywords:** milk fat, fatty acids, Lipizzaner mares, lactation

## Abstract

The composition and properties of the milk fat of mares’ milk is interesting from a nutritional standpoint. The aim of this study was to determine the nutritional value of Lipizzaner mare’s milk for possible human consumption and identity the influence of the parity and stage of lactation on its fatty acid (FA) composition. This study was conducted on 17 Lipizzaner mares from a state stud farm in the Slavonian region (eastern Croatia). Mares were hand-milked twice during lactation in the fourth and sixth months. Significantly higher contents of MUFAs (monounsaturated fatty acids) and PUFAs (polyunsaturated fatty acids) and lower ratios of n-6/n-3 were found in the fourth month of lactation. This parity significantly affected the PUFA/SFA ratio (polyunsaturated fatty acids/saturated fatty acids), with lower values found in older mares. The fatty acid composition of mare’s milk that is especially high in UFAs (unsaturated fatty acids) is considered to be beneficial for consumers.

## 1. Introduction

Scientific findings have highlighted the potential use of equine milk as a beneficial food for human health regarding its protein and lipid composition [1].

There is a growing tendency worldwide to prefer equine milk over the milk of other animals [1]. Approximately 30 million people regularly consume mare’s milk or koumiss, and this number is steadily growing [2]. The diverse composition and properties of mare’s milk fat make it interesting from a nutritional point of view [3]. The unique composition and health-promoting properties of mare’s milk could lead to higher consumption [4].

The fatty acids (FAs) in milk fat are considered to be important nutritional components of human diets and substantially affect human health. The fat content of mare’s milk is very low compared to human’s and cow’s milk [5,6].

Although the fat content of mare’s milk is low, it is characterized by a high polyunsaturated fatty acid (PUFA) content [1]. Mare’s milk is characterized by high quantities of lactose (from 58 to 70 g kg^−1^) and protein (from 15 to 28 g kg^−1^) and a reduced fat content (from 3 to 20 g kg^−1^) [7]. The diameter of mare’s milk fat globules is 2–3 μm [8]. Human’s, cow’s, and mare’s milk triglycerides account for approximately 80% of the total lipids; phospholipids and sterols account for 5%; and free fatty acids account for 10% of the total lipids [9]. Cholesterol in mare’s milk ranges from 50 to 88 mg L^−1^ [10,11,12]. Milk fat is a remarkable source of energy, fat-soluble nutrients, and bioactive lipids [13].

The immunonutrients in milk must also be considered; these are primarily vitamins and long-chain PUFAs that regulate the immune function and may be involved in the development and severity of inflammation symptoms [14]. The profile of FAs largely determines the health quality of milk. To assess the nutritional value for consumer health, the n-6/n-3 ratio, atherogenic index (AI), and thrombogenic index (TI) are commonly used as important determinants of reduced risk of coronary heart disease and cancer [12]. In addition, several studies have demonstrated the beneficial effects of dietary PUFAs on neuronal functions [15].

Low-fat dairy products, when consumed regularly as part of a balanced diet, may have several beneficial outcomes for neurocognitive health [16]. The development of different diseases, including cardiovascular and Alzheimer’s disease, may also be affected by higher proportions of n-6 compared to n-3 PUFAs in the diet [17]. The production of mare’s milk and derivatives with high nutritional value and health-promoting properties should, therefore, be considered as a promising extension of equine management for contemporary and future society [18].

The relationship between prothrombogenic (saturated fatty acids, SFAs) and antithrombogenic fatty acids (monounsaturated fatty acids, MUFAs; n-3 and n-6 polyunsaturated fatty acids, PUFAs) is interesting. Serum n-6 PUFAs exhibit atherosclerotic effects while n-3 PUFAs inhibit platelet aggregation and lower cholesterol and phospholipids [19]. Saturated fatty acids are the most represented class in mare’s milk compared to MUFAs or PUFAs [20]. Fatty acid variability is most likely related to dietary and/or body condition differences, as reviewed for monogastric herbivores [21]. A promising area has opened in which the possibility of direct fermentation of mare’s milk is provided. Mare’s milk, compared with cow’s milk, has much lower values of the AI and TI. The hypocholesterolemic/hypercholesterolemic ratio and n-6/n-3 ratio values are also favorable, which are particularly low at approximately 0.9 [22]. Unfermented and fermented equine milk is a source of many advantageous ingredients [23]. According to Garcia et al. [23], the total phospholipid content was higher in camel’s (0.503 mM) and lower in mare’s (0.101 mM) compared to human’s (0.324 mM) or cow’s (0.265 mM) milk. According to the presence of long-chain highly unsaturated fatty acids in mare’s milk and evidence of the close similarity between human and equine milk fat globule membrane proteins in the unique fatty acid composition, the use of mare’s milk is supported for human nutrition [10,14]. The study by Czyżak-Runowska [24] indicated a significant correlation between the number of foalings and the FA profile. Mare’s milk had an average PUFA n6:n3 ratio of 0.57 to 1.40 while in human’s milk, the ratio between proinflammatory (derived from n6 long-chain PUFAs) and anti-inflammatory (derived from n3 long-chain PUFAs) immunonutrients was reported to vary from 5.1 to 11.2 [20,25,26]. PUFAs (<0.5 g 100 g1 fatty acids) in mare’s milk contain eicosapentaenoic (EPA, C20:5 n3) and docosahexaenoic (DHA, C22:6 n3) FAs [21,27], whose roles in the development of the neonatal brain and retina [24] and inflammatory response pathways [28,29] have been reported.

The aim of this study was to present the nutritional value of Lipizzaner mare’s milk for human consumption and to determine the effect of the parity and stage of lactation on the fatty acid (FA) composition.

## 2. Results

### 2.1. Basic Chemical Composition of Milk

The basic components of mare’s milk are presented in Table 1. The average values of fat and protein were 1.18% and 1.53%, respectively. The average percentage of lactose was 6.56% while the solids-not-fat (SNF) were 9.01%. The range of fat was from 0.25 to 3.04%, and the SNF ranged from 8.67 to 9.21%. The values of lactose and proteins ranged from 5.71 to 6.88% and from 1.30 to 2.04%, respectively.

The ranges of all analyzed fatty acids in mare’s milk are presented in Table 2. These ranged from a minimum of 0.01 to a maximum of 60.08. The lowest average values were recorded for C20:1 (0.28), C20:4 n-6 (0.67), C17:0 (0.38), and C9:0 (0.59), and the highest average values were recorded for C16:0 (21.66) and C18:1 (31.03) (*n* = 17 × 2).

In Table 3, the composition of fatty acids and the lipid indexes of mare’s milk fat are presented. In Lipizzaner mare’s milk, more unsaturated fatty acids (UFAs) were established compared to saturated fatty acids (SFAs) (57.21:43.78), and these ranged from 10.94 to 76.60 and 23.40 to 89.05, respectively. The average values of the monounsaturated (MUFA) and polyunsaturated (PUFA) fatty acids ranged from 2.58 to 52.55, with average values of 30.34 and 26.87%, respectively. As for the relationship between the individual acids, the highest values were recorded for SCD:18 (sterol-CoA desaturase) at 87.11, and the lowest were recorded for PUFA:SFA at 0.79 (*n* = 17 × 2).

### 2.2. Influence of the Parity and Stage of Lactation on the Chemical Composition of Mare’s Milk

The basic composition of the mare’s milk according to the parity and stage of lactation is presented in Table 4. The basic milk composition was stable through parity, with slightly insignificantly lower values in older mares (over third lactation). As opposed to parity, significant influences were found for the stage of lactation as follows: the composition of fat in mare’s milk was significantly (*p* < 0.0002) higher in the fourth compared to the sixth month of lactation (1.66:0.70). Toward the end of lactation (sixth month), the total amount of milk fat in mare’s milk decreased.

In this study, the lactose content was around 6.70 for all mares. There was a significant (*p* < 0.0003) difference according to the stage of lactation: the mares in the fourth month had a significantly lower value of lactose compared to the mares in the sixth month of lactation (6.43: 6.69). There was no significant variability in the composition of lactose and protein in mare’s milk owing to parity. The analyzed average fat content in the periods of lactation was stable, and it ranged from 0.70 to 1.66. The lowest value of fat in the mare’s milk was 0.70 in the sixth month of lactation, and a significant (*p* < 0.0002) difference was detected.

**Figure 1:**
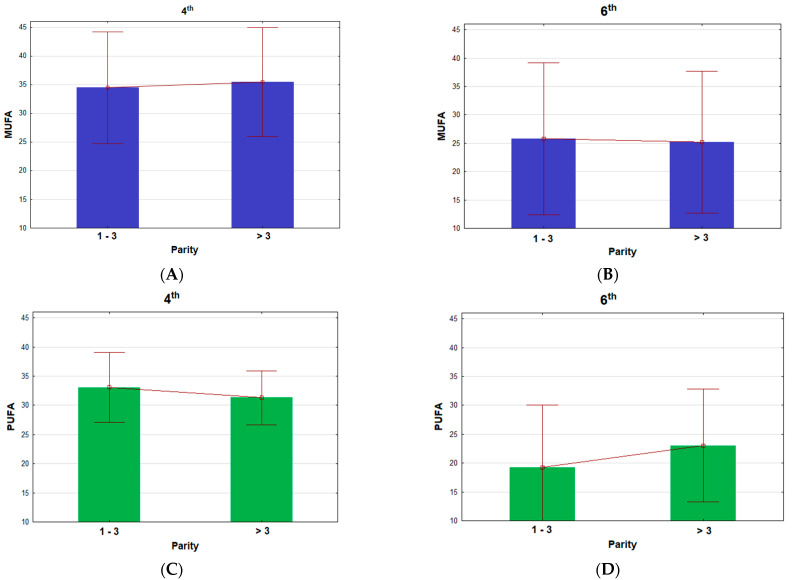
Fatty acids (% of total lipids) according to the parity and stage of lactation (in the fourth month (**A**,**C**); in the sixth month (**B**,**D**)). MUFAs; C10:1, C12:1, C14:1 cis-9, C16:1 cis-9, C16:1 trans-9, C17:1 cis-9, C18:1 cis-9, C18:1 cis-11, C18:1 cis-12, C18:1 trans-9, C18:1 trans-11, and C20:1 cis-11, PUFA; C18:2 cis-9, trans-11, C18:2 cis-9, cis-12, C18:3 cis-6, cis-9, cis-12, C18:3 cis-9, cis-12, cis-15, C20: 2n−6, C20: 3n−6, C20: 4n−6, C20: 5n−3, and C22: 5n−3.

Figure 1 shows the variable relationship of MUFAs and PUFAs according to the parity and stage of lactation. The values of PUFAs were over 32% in the fourth month and over 19% in the sixth month of lactation. The MUFA values were over 34 and 25% in the fourth and sixth months, respectively. The contents of PUFAs were 5% lower than those of MUFAs. A reverse trend of the MUFA and PUFA values was determined between lactation stages depending on the parity, as follows: MUFAs were higher and PUFAs were lower in older mares in the fourth month of lactation, but the opposite occurred in the sixth month where the MUFAs were lower and PUFAs were higher in older mares. The PUFA fatty acid groups were significantly (*p* < 0.003) affected by the stage of lactation (Table 5).

Table 5 illustrates that saturated fatty acid (SFA) values in milk fat were influenced by the stage of lactation. The SFA level in the sixth month was significantly (*p* < 0.002) higher than in the fourth month of lactation. Parity did not have a significant effect on SFAs. In the present study, the SFA values were 53.43% of the fatty acids, and the SFA content increased during the course of lactation.

In the present study, the unsaturated fatty acid (UFA) values were 46.65–67.18% for total lipids (Table 5). The UFA values were statistically significant (*p* < 0.002) regarding the stage of lactation, and the total value was lower in the sixth month of lactation. The UFA value in the sixth month of lactation decreased by 20.53 compared to the fourth month. Parity did not significantly influence the composition of UFAs in the mare’s milk.

Of the total amount of fatty acids monitored, the mean content of MUFAs (monounsaturated) was 25.50–34.91%. The MUFA content tended to gradually decrease in the sixth month of lactation. The MUFA level was lower in the sixth month than in the fourth month of lactation. Parity had no statistically significant effect on the MUFAs.

There were no big changes in the composition of n-3 FAs according to the parity or stage of lactation. The values of n-3 FAs in mare’s milk ranged between 8.28 and 8.80. There were significant changes in the composition of n-6 FAs in this study with respect to the lactation stages. The amount of n-6 in the sixth month of lactation was significantly (8.87; *p* < 0.006) lower than in the fourth month of lactation. Parity did not significantly influence the changes in the amount of n-6 FAs.

The n-6/n-3 ratio ranged between 1.84 and 3.54, without a significant change according to parity. The stage of lactation had a significant influence, and mares in the sixth month had significantly lower values compared with those in the fourth month of lactation (1.84:3.54; *p* < 0.04). The SCDi16 ratio ranged between 17.17 and 13.20; a significant influence was exhibited between the fourth (17.17) and sixth (13.20) months of lactation (*p* < 0.02), with a decrease of 3.97. There was no significant difference with regard to parity. The ratio of SCDi18 ranged between 83.31 and 91.69 in relation to the number of lactating mares and was not as significant as for lactation herds. C18-1-C18-0 showed constant values between 13.66 and 14.88 and were independent of the mare lactation number and lactation stage. The PUFA values in the mare’s milk were statistically significant (*p* < 0.003) in the stage of lactation; PUFA values in the fourth month were 11.11 higher than in the sixth month of lactation. No statistical difference was found for parity.

The PUFA n6:n3 ratio averaged 1:1. PUFAs changed according to the stage of lactation in the fourth and sixth months, with a decrease in the value of 21.16% in the sixth month for n-6 FAs. In this study, the PUFA ratio ranged from 32.27 to 21.16. The ratio of n-6/n-3 ranged from 3.54 to 1.48; it was advantageous in the sixth month of lactation. In this study, statistically significant values of PUFA:SFA were determined in parity. Mares in one to three lactations had a higher value for PUFA:SFA by 0.91 (2.16:1.25; *p* < 0.002). The PUFA: SFA ratio was statistically significantly affected (*p* < 0.001) by the stage of lactation and was 0.44 lower in the sixth month of lactation compared to the fourth month (1.00:0.56; *p* < 0.001). Furthermore, statistically significant values of UFA:SFA were found between lactations (*p* < 0.002). In the sixth month of lactation, the value of UFA:SFA decreased by 0.76 compared to the fourth month of lactation. There were no statistical differences in UFA:SFA with regard to parity. High contents of both linoleic (C 18:2 n6) and linolenic (C 18:3 n3) acids were established for mare’s milk. The contents of different fatty acids in the fat of mare’s milk varied in this study.

## 3. Discussion

Mare’s milk differs greatly from the milk of other mammal species in terms of the component contents (fat, protein, lactose, and solids-not-fat) [30]. Characteristic features of mare’s milk include low contents of fat and proteins and a high content of lactose, which can also be seen in this study (Table 1), and toward the end of lactation, the total amount of milk fat in mare’s milk decreased (Table 4). The fat content in the present study was similar to the value reported by Czyżak-Runowska et al. [24]. In the sixth month of lactation, in this study, the percentage of fat in mare’s milk was lower compared to the values reported by Barreto et al. [1]. The lactose concentration of mare’s milk was relatively high in comparison with milk from cows, goats, and sheep [20,31]. A high lactose content is characteristic of mare’s milk and is constant throughout lactation. The constant sweet taste due to the lactose gives mare’s milk a recognizable taste [7,30,32,33,34]. The lactose concentration in this study was affected by the stage of lactation and was higher in the sixth month, but no influence of parity was noted.

The quantitative and qualitative ratio of FAs ingested in food is important and, to a great extent, determines the composition and functions of lipids in the consumer’s body. Digested dietary fats, soluble carbohydrates, proteins, and minerals (except for escaped nutrients and part of phosphorus) are mainly absorbed by the small intestine of the horse; the scarce or null biohydrogenation before absorption suggests the direct influence of diet on the FA composition of milk, as previously suggested for human’s milk [26]. The percentages of UFAs in mare’s and human’s milk are similar and higher than that in cow’s milk. This is mainly due to the high content of PUFAs. The content of different fatty acids in the fat of mare’s milk varies regarding horse breed [30]. In the present study, the UFA values were 46.65–67.18% of the total lipids and were in accordance with those found by Salimei and Fantuz [20] and the PUFAs detected by Pietrzak-Fiecko et al. [35] for the Wielkopolski horse. The ratio of unsaturated-to-saturated fatty acids in Lipizzaner mare’s milk in the present study was 1.7:1 and was similar to human’s milk, with 20% linoleic acid and 8% α-linolenic acid (Table 2). The average value of PUFAs in this study was over 20%, with the lowest value in the sixth month of lactation. In the studies by Navrátilová et al. [3] and Barello et al. [10], PUFA levels were lower than in the present study. Czyżak-Runowska et al. [24] reported a higher PUFA and MUFA content in the sixth month of lactation. The PUFAs are precursor molecules of long-chain polyunsaturated fatty acids, structural components of all cellular membranes and precursors of eicosanoids, molecules that modulate various cellular and tissue processes.

With regard to the milk’s FA composition, the SFA content was lower compared to UFAs in mare’s milk, although wide variability can be observed in the experimental data. The content of different FAs in the fat of mare’s milk varied in this study and in [26,36,37,38]. Mare’s milk stearic acid (C18:0) content was very low compared to human’s and cow’s milk [11].

The saturated fatty acid values in milk fat were influenced by the stage of lactation (Table 2), with higher values in the sixth month of lactation. The results agreed with those obtained by Navrátilová et al. [3] and are opposite to the findings of Czyzak-Runowska et al. [24]. It is recommended that the consumption of n-6 should be balanced with that of n-3 PUFA, with a ratio between 1:1 and 5:1.2 [39].

The MUFA content in mare’s milk was lower than in human’s milk [3,5,11]; in the present study, it was not affected by the parity or lactation stage. It is recommended that the consumption of n-6 FAs should be balanced with that of n-3 PUFAs, with a ratio between 1:1 and 5:1.2 [39]. The high levels of total n-3 PUFAs were well balanced by the total n-6 PUFA content of mare’s milk. The variability in mare’s milk is related to dietary and/or body condition differences, as reviewed for monogastric herbivores [40,41]. The values of n-3 FAs in mare’s milk ranged between 8.28 and 8.80, which are lower than those reported by Teichert et al. [22]. The content of n-6 FAs in this study was affected by the lactation stage, with lower values in the sixth month of lactation, which makes mare’s milk even more favorable than human’s milk in the sixth month of lactation. The measured values were a few times higher than in human’s and cow’s milk [11].

The n-6/n-3 ratio in the present study ranged from 3.1:1 to 1.8:1, which makes mare’s milk a good source for human consumption. This showed no significant change regarding parity. A significant influence was established regarding the stage of lactation, with lower values in the sixth month of lactation. These values are similar to those identified in the studies of Navrátilová et al. [3] and Barello et al. [10].

The percentages of unsaturated fatty acids in mare’s and human’s milk are similar and higher than those in cow’s milk. This is due mainly to the high content of polyunsaturated fatty acids (PUFAs), with intermediate and high numbers of carbon atoms, as in this study, the PUFA n6:n3 ratio averaged 1:1. PUFAs changed according to the stage of lactation (by 21.16%). It has been recommended that the consumption of n-6 should be balanced with that of n-3 PUFAs, with a ratio between 1:1 and 5:1.2 [39]. The high levels of total n3 polyunsaturated fatty acids (PUFAs) were well balanced by the total n6 PUFA content of mare’s milk. The variability in mare’s milk is related to dietary and/or body condition differences, as reviewed for monogastric herbivores [5,21,40,41,42]. This was not demonstrated in this study because mares of the Lipizzaner breed are equally fed. The mare’s milk was in fact characterized by a PUFA n6: n3 ratio that averaged between 0.57 and 1.40 while this ratio between proinflammatory (derived from n6 long-chain PUFAs) and anti-inflammatory (derived from n3 long-chain PUFAs) immunotargets is reported to vary from 5.1 to 11.2 in human’s milk [25,26]. In this study, the PUFA ratio ranged from 32.27 to 21.16. The ratio of n-6/n-3 ranged from 3.54 to 1.48, and it is advantageous that in the sixth month of lactation, the value was lower because the mares were milked to provide milk for humans. In this study, mares in one to three lactations had a significantly higher PUFA value than SFAs by 0.91 (2.16:1.25; *p* < 0.002). Furthermore, the PUFA:SFA ratio was statistically significantly affected (*p* < 0.001) by the stage of lactation and by parity (*p* < 0.002). In the sixth month of lactation, the value of UFA:SFA decreased by about 0.76 compared to the fourth month of lactation. There were no statistical differences in UFA:SFA with regard to parity. There was an increase in the saturated fatty acids with a parallel decrease in the concentration of monounsaturated and polyunsaturated fatty acids [39]. Digested dietary fats, soluble carbohydrates, proteins, and minerals (except for escaped nutrients and part of phosphorus) are mainly absorbed by the small intestine of the horse; the scarce or null biohydrogenation before absorption suggests the direct influence of diet on the fatty acid composition of milk, as previously suggested for human’s milk [26]. In addition to the dietary factors, the low stearic acid (C18:0) content of mare’s milk is important to note. In previous research [8], there were no significant deviations from this study regarding the C18-1-C18-0 values from 14.74 to 13.66, which are explained by the D9 desaturase activity in the mammary gland. High contents of both linoleic (C 18:2 n6) and linolenic (C 18:3 n3) acids were reported for mare’s milk. In this study, according to literature data [23,30,36,41,42,43,44,45], the content of different fatty acids in the fat of mare’s milk varied.

## 4. Materials and Methods

### 4.1. Experimental Design

Mare’s milk was collected from 17 Lipizzaner mares reared on a state stud farm in the Slavonia region (eastern Croatia). The mares had the following body measurements: body weight between 450 and 535 kg and wither height between 156 and 162 cm. All mares were fed the same feed. During the day, for 13 h, all mares were on pasture with meadow grass. In addition to grazing, they received 4.5 kg of oats daily and hay ad libitum. During the experiment, all mares were clinically healthy.

The mares were hand-milked from both teats of the udder once a day in the morning after 3 h of physical separation from their foals. The mares were milked in the fourth and sixth months of lactation (stage of lactation). Lipizzaner mares were classified into two groups: (A) younger (from first to third lactation, *n* = 8) and (B) older (over third lactation, *n* = 8).

### 4.2. Equipment

The solids-not-fat, fat, lactose, casein, whey protein, and ash contents were determined using a MilkoScan™ FT120 (Foss Tecator AB Hilleroed, Denmark).

### 4.3. Extraction of Total Lipids

Total lipids were extracted with a modified method of Folch et al. [45] using a solvent mixture of chloroform: methanol: water. The final ratio of extraction solvent was chloroform: methanol: water = 2:2:1.8. Extracts of total lipids were evaporated in a UNIVAPO 100H evaporator, equipped with a UNICRYO MC 2L Uniequip refrigeration unit (Uniequip, Planegg, Germany). Total lipid extracts were concentrated in a UNIVAPO 100H rotary evaporator, equipped with a UNICRYO MC 2L cooling unit (Uniequip, Planegg, Germany). Total lipid extracts were stored at −20 °C until analysis.

### 4.4. Preparation of Fatty Acid Methyl Esters

Fatty acids from the total lipid extract were converted to methyl esters trans-esterification according to the international standard procedure ISO 14156-IDF 172:2001 and ISO 15884-IDF 182:2002. The resulting methyl esters of fatty acids were prepared for analysis with gas chromatography. In the sample of the total lipid, methyl nonadecanoic acid (C19:0) was used as an internal standard

### 4.5. Gas Chromatography (GC)

Analysis of fatty acid methyl esters was performed with a gas chromatograph (Agilent 8860; Agilent Technologies. Inc., Santa Clara, CA, USA) equipped with a flame ionization detector (FID) and ALS. The temperature of the injector was 200 °C and the temperature of the detector was 240 °C. Chromatography was performed on a capillary column DB-23 (Agilent Technologies, Santa Clara, CA, USA), length 60 m, inner diameter of the column 0.25 mm, active layer thickness 0.25 μm). The initial column temperature was 150 °C for 2 min; it was then increased to 230 °C by heating at 5 °C/min and held at that temperature for 20 min. Hydrogen at a flow rate of 1 mL/min was used as the carrier gas. Collection and processing of the results were conducted with the computer program OpenLAB CDS ChemStation Workstation VL. Fatty acids were identified by comparing the retention times with methyl standards (Sigma Aldrich Chemie, GmbH and Supelco, St. Louis, MI, USA). Quantification was performed using nonadecanoic acid methyl ester (C19:0). The fatty acid composition was calculated as the percentage of each individual fatty acid relative to the total fatty acids.

### 4.6. Statistical Analysis

For the data preparation and statistical analysis, SAS software (SAS Institute Inc., Cary, NC, USA, 2019) was used. According to parity, mares were classified into two age classes: younger, animals in the first, second, and third lactation; and older, animals in the fourth and higher lactation). Furthermore, according to the stage of lactation, records were divided into two lactation month classes (fourth, sixth). The variability in the analyzed traits due to age classes (younger, from first to third lactation; and older, from fourth lactation) and lactation month (fourth, sixth) was tested using least-square means in a GLM (Generalized Linear Model) procedure in SAS (SAS Institute Inc., Cary, NC, USA, 2019). The following statistical model was used:

$$y_{ijk}=\mu +A_{i}+L_{j}+e_{ijk}$$

where:

*y_ijk_* = estimated trait.

*μ* = intercept.

*P_j_* = fixed effect of age *i* (*i* = younger (from first to third lactation); older (from fourth lactation)).

*L_j_* = fixed effect of lactation month j (j = fourth; sixth).

*e_ijk_* = residual.

Tukey’s test in PROC GLM (SAS) was used to determine the significance (*p* < 0.05) of the differences in the analyzed traits according to age class and location months.

## 5. Conclusions

The results of this research present a new aspect of Lipizzaner horse breeding with additional economic value. The analysis of the composition of Lipizzaner mare’s milk determined its suitable nutritional composition as a functional food for human nutrition. These results indicate the possible commercial production of mare’s milk from this breed. A more favorable nutritional composition was found in the fourth month compared to the sixth month of lactation. Results showed that parity had no effect on milk FA composition, which could be advantageous in terms of the mare’s production life. The results from this research reveal the potential for the greater consumption of mare’s milk by humans. Because of its unique FA composition, mare’s milk and its derivatives could become valuable foods for elderly consumers. Specific milk-processing technologies are needed to improve the shelf life of mare’s milk, preserving both the natural attributes of mare’s milk and the health of sensitive consumers.

## Figures and Tables

**Table 1:** The chemical composition of mare’s milk.

Variable	Mean	Min	Max	SD	CV	SE
Solids-not-fat (%)	9.01	8.67	9.21	1.13	1.46	0.02
Fat (%)	1.18	0.25	3.04	0.78	66.03	0.14
Lactose (%)	6.56	5.71	6.88	0.22	3.33	0.04
Protein (%)	1.53	1.30	2.04	0.14	9.15	0.02

**Table 2:** The fatty acid composition of mare’s milk (g/100 g total fatty acids).

FA	Mean	Min	Max	SD	CV	SE
C8:0	1.24	0.12	4.07	1.27	102.21	0.28
C9:0	0.59	0.11	1.90	0.56	94.32	0.14
C10:0	1.70	0.14	8.78	1.87	110.06	0.36
C12:0	2.65	0.21	10.35	2.55	96.05	0.46
C14:0	9.29	0.24	60.08	13.16	141.69	2.36
C15:0	3.86	1.11	10.21	2.40	62.15	0.42
C16:0	21.66	9.42	40.60	6.27	28.95	1.09
C16:1	4.02	0.36	7.87	1.80	44.85	0.32
C17:0	0.38	0.07	0.68	0.14	36.98	0.03
C18:0	3.40	0.36	10.97	2.08	61.23	0.36
C18:1	31.03	0.01	38.82	9.81	31.62	1.85
C18:2 n-6	19.92	1.71	47.74	8.78	44.07	1.60
C18:3n-3	8.24	2.50	16.03	3.04	36.92	0.53
C20:1	0.28	0.12	0.51	0.17	44.66	0.03
C20:4 n-6	0.67	0.20	2.49	0.52	77.96	0.10
n-6	19.84	1.47	47.74	9.36	47.18	1.68
n-3	8.53	2.50	17.03	3.40	39.86	0.59

**Table 3:** The values of healthy fatty acids and ratios between fatty acids in mare’s milk.

FA	Mean	Min	Max	SD	CV	SE
SFA	43.78	23.40	89.05	19.81	46.31	3.45
MUFA	30.34	2.58	43.12	14.02	46.20	2.44
PUFA	26.87	4.48	52.55	11.06	41.17	1.92
UFA	57.21	10.94	76.60	19.81	34.63	3.45
UFA:SFA	1.72	0.12	3.27	0.90	52.61	0.16
PUFA:SFA	0.79	0.05	1.49	0.41	52.09	0.07
C18:1/C18:0	14.19	0.00	73.53	13.03	91.80	2.46
n-6:n-3	2.76	0.11	11.32	2.36	84.59	0.42
SCD:16	15.18	3.69	24.37	4.99	32.86	0.88
SCD:18	87.11	0.21	98.66	18.74	21.51	3.54

Note. SFAs—sum of saturated fatty acids (C8:0, C9:0; C10:0, C12:0, C14:0, C15:0, C16:0, C17:0, C18:0); UFAs—sum of unsaturated fatty acids (MUFA; C10:1, C12:1, C14:1 cis-9, C16:1 cis-9, C16:1 trans-9, C17:1 cis-9, C18:1 cis-9, C18:1 cis-11, C18:1 cis-12, C18:1 trans-9, C18:1 trans-11, and C20:1 cis-11); polyunsaturated fatty acids (PUFAs; C18:2 cis-9, trans-11, C18:2 cis-9, cis-12, C18:3 cis-6, cis-9, cis-12, C18:3 cis-9, cis-12, cis-15, C20: 2n−6, C20: 3n−6, C20: 4n−6, C20: 5n−3, and C22: 5n−3); n-3 FAs—sum of C18: 3n−3, C20: 5n−3, and C22: 5n−3; n-6 FAs—sum of C18: 2n−6, C18: 3n−6, C20: 2n−6, C20: 3n−6, and C20: 4n−6; SCDi16 Activity indexes of the sterol-CoA desaturase (SCD) SCDi-16 = [16:1/(16:1 + 16:0)] × 100; SCDi18; Activity indexes of the sterol-CoA desaturase (SCD) SCDi-18 = [18:1/(18:1 + 18:0)] × 100. SE, standard error of the mean (*n* = 17 × 2).

**Table 4:** Gross composition of mare’s milk.

Variable	Parity					Stage of Lactation				
	1–3 (*n* = 9)	SD	>3 (*n* = 8)	SD	*p*	4th (*n* = 17)	SD	6th (*n* = 17)	SD	*p*
Solids-not-fat (%)	9.02	0.13	9.00	0.14	0.68	8.99	0.15	9.03	0.11	0.43
Fat (%)	1.20	0.94	1.16	0.57	0.85	1.66 ^a^	0.66	0.70 ^b^	0.57	0.0002
Lactose (%)	6.61	0.26	6.61	0.15	0.11	6.43 ^a^	0.24	6.69 ^b^	0.08	0.0003
Protein (%)	1.60	0.17	1.49	0.08	0.09	1.51	0.18	1.53	0.10	0.95

**Table 5:** LSMs of the composition of healthy fatty acid and ratios between fatty acids in mare’s mil.

FA	Parity					Stage of Lactation				
	1–3 (*n* = 9)	SD	>3 (*n* = 8)	SD	*p*	4th (*n* = 17)	SD	6th (*n* = 17)	SD	*p*
SFA	43.65	21.62	42.50	18.40	0.85	32.81 ^a^	6.78	53.34 ^b^	23.61	0.002
UFA	56.35	21.60	57.50	18.37	0.85	67.18 ^a^	6.76	46.65 ^b^	23.79	0.002
MUFA	30.10	14.57	30.31	13.89	0.96	34.91	11.70	25.50	14.99	0.06
n-3 FA	8.38	3.57	8.70	3.32	0.80	8.28	2.85	8.80	3.97	0.67
n-6 FA	20.38	10.53	18.49	8.22	0.54	23.82 ^a^	6.73	14.95 ^b^	6.84	0.006
n-6/n-3	3.13	3.13	2.26	1.21	0.28	3.54 ^a^	2.74	1.84 ^b^	1.34	0.04
SCDi16	15.11	4.90	15.24	5.23	0.94	17.17 ^a^	4.67	13.20 ^b^	4.60	0.02
SCDi18	91.69	5.19	83.31	24.90	0.26	86.62	23.35	88.07	10.86	0.87
C18-1-C18-0	13.66	5.68	14.74	17.30	0.83	13.88	6.28	14.51	19.04	0.90
PUFA	26.24	12.40	27.19	9.84	0.79	32.27 ^a^	6.67	21.16 ^b^	12.09	0.003
PUFA: SFA	2.16 ^a^	0.43	1.25 ^b^	0.40	0.002	1.00 ^a^	0.22	0.56 ^b^	0.45	0.001
UFA: SFA	1.72	0,91	1.70	0.92	0.99	2.16 ^a^	0.61	1.24 ^b^	0.94	0.002

Note. SFAs—sum of saturated fatty acids (C8:0, C9:0; C10:0, C12:0, C14:0, C15:0, C16:0, C17:0, C18:0); UFA—sum of unsaturated fatty acids (MUFAs; C10:1, C12:1, C14:1 cis-9, C16:1 cis-9, C16:1 trans-9, C17:1 cis-9, C18:1 cis-9, C18:1 cis-11, C18:1 cis-12, C18:1 trans-9, C18:1 trans-11, and C20:1 cis-11); polyunsaturated fatty acids (PUFAs; C18:2 cis-9, trans-11, C18:2 cis-9, cis-12, C18:3 cis-6, cis-9,cis-12, C18:3 cis-9,cis-12,cis-15, C20: 2n−6, C20: 3n−6, C20: 4n−6, C20: 5n−3, and C22: 5n−3); n-3 FAs—sum of C18: 3n−3, C20: 5n−3, and C22: 5n−3, n-6 FAs—sum of C18: 2n−6, C18: 3n−6, C20: 2n−6, C20: 3n−6, and C20: 4n−6; SCDi16 Activity indexes of the sterol-CoA desaturase (SCD) SCDi-16 = [16:1/(16:1 + 16:0)] × 100; SCDi18 Activity indexes of the sterol-CoA desaturase (SCD) SCDi-18 = [18:1/(18:1 + 18:0)] × 100. SE, standard error of the mean.

## Data Availability

The data presented in this study are available on request from the corresponding author. The data are not publicly available due to data comes from a state stud farm, which does not allow open access.
